# Rooting, Growth, and Root Morphology of the Cuttings of *Ficus carica* L. (cv. “Dottato”): Cutting Types and Length and Growth Medium Effects

**DOI:** 10.3390/plants14020160

**Published:** 2025-01-08

**Authors:** Rocco Mafrica, Marcello Bruno, Vincenzo Fiozzo, Roberta Caridi, Agostino Sorgonà

**Affiliations:** 1 Department of AGRARIA, University “Mediterranea” of Reggio Calabria, 89124 Reggio Calabria, Italy; vincenzofiozzo@yahoo.it (V.F.); roberta.caridi@unirc.it (R.C.); asorgona@unirc.it (A.S.); 2 Calabrian Agriculture Development Regional Agency (A.R.S.A.C.), 87100 Cosenza, Italy; marcello.bruno@arsac.calabria.it

**Keywords:** cutting type, rooted cutting, fig propagation, root morphology, multivariate analysis

## Abstract

The fig tree (*Ficus carica* L.) has gained renewed interest for its climate resilience and the health benefits of its fruit, driving demand for high-quality nursery plants. However, suboptimal propagation techniques limit the nursery production of figplants. This study evaluated the influence of the type and length of the cutting and the growth medium on rooting success, biomass yield and allocation, and root morphology in fig plants of the “Dottato” cultivar. Results pointed out that distal and longer cuttings significantly enhanced rooting efficiency and biomass production and allocation, yielding optimal shoot and root morphology for transplanting success. Multivariate analysis identified critical traits differentiating fig nursery plants’ quality across treatments. Additionally, the results showed that favorable outcomes were achieved across various growth mediums. These insights provide practical strategies to optimize propagation techniques and improve plant quality for sustainable fig cultivation.

## 1. Introduction

The fig (*Ficus carica* L.) is a deciduous fruit tree native to southwest Asia and the eastern Mediterranean. It has been widely cultivated since ancient times for the nutritional value of its fruits, commonly referred to as figs. Generally, fig trees grow best and produce high-quality fruit in the Mediterranean basin [1], which accounts for over 80% of the world’s fig production, with Italy being the second-largest fig-producing country in the European Union with an average annual production of around 9500 tons [2]. Nowadays, as a climate-resilient plant species, the fig is being cultivated extensively in many regions of the world due to its higher adaptability to variable climatic and soil types and its minimum signs of deficiencies or toxicity symptoms [3]. Being both a climate-resilient species and a source of health benefits through its fruits [4], the market demand for fresh and dried figs has increased [5], encouraging fig growers to establish new fig plantations. However, the high demand for fig plants has not been satisfied by fig nurseries due to poor organization and, above all, a lack of improvement and optimization in propagation techniques.

The fig is usually propagated by various asexual methods, such as burrs, plunging, grafting, and tissue culture. However, these methods are unsustainable for large-scale use due to the laboriousness of the operations and the relatively high costs compared to propagation by cuttings, which is the most commercially used method [6]. This method involves inducing adventitious roots in segments of the mother plant under specific environmental conditions [7,8]. Hence, the success of cutting propagation in fruit crops depends upon many crucial factors such as the physiological condition and age of the mother plant, harvest time, lignification status, use of plant regulators, type of rooting media, and cultivation environment [6], as well as the different rooting ability of each plant species [9]. In this context, the aim of this study was to evaluate the effects of cutting type and length on the rooting percentage of the fig variety Dottato, the most traditional fig variety in the Calabrian region (south of Italy) [10], which showed the richest and most diversified Italian fig germplasms [11,12,13]. The Dottato (synonyms: ‘Fico bianco’ and ‘Kadota’) develops parthenocarpic fruits that do not require pollination to bring the syconia to maturity. Although it is a two-crop variety, the breba crop is very limited. The main production is represented by figs in southern Italy that ripen in the second half of summer. The Dottato is primarily used for producing dried figs (its name derives from the Greek ‘optao’ meaning “to dry”), but it is also consumed fresh or used in other processed products [10]. In Italy, it is the fig variety authorized for producing two protected designations of origin (PDO): “Fichi di Cosenza” and “Fico bianco del Cilento”.

In addition to uniformity and health status, the quality of nursery fruit plants is based solely on their rooting ability in terms of both size and time, without considering that root morphology is crucial for successful transplantation in the field [14]. This *modus operandi* has also been adopted for the fig tree. In fact, most studies on fig nursery plants have mainly focused on a few morphological parameters of the roots, mainly length and the number and percentage of root emission [6,8,15,16,17,18,19]. This limited approach overlooks several root morphological traits, known as functional traits or “phenes”, which are associated with plant ecological and growth roles, such as water and nutrient uptake, root length and surface area [20,21,22], the root length ratio (RLR, the root length per unit of the plant’s dry mass) [23], the root mass ratio (RMR, root dry mass per unit of the plant’s dry mass) [22], root fineness (RF, root length per unit root volume) [22,24,25], root tissue density (RTD, root dry mass per unit root volume) [21], and the length of very fine, fine, and coarse roots [22]. Only two studies deeply investigated root morphology when cuttings were treated with different AMF formulations [26,27].

In this study, the effects of the cutting type and length on the root morphology of fig root cuttings have been analyzed, considering the importance of root traits as predictors of transplant success [14,28]. Additionally, the impact of the growth medium on the root system [29] was evaluated. Two different growth mediums, perlite and a soil/peat/sand mixture, were included in the experimental setup to assess their effects on root morphology and the growth of fig root cuttings.

## 2. Materials and Methods

### 2.1. Experimental Site and Cutting Collection and Preparation

This research was carried out at a nursery located in Bisignano (South Italy) (altitude of 118 m above sea level, latitude 39°31′09.39″ N and longitude 16°14′49.36″ E). Woody stems used for cuttings were collected in late January from ten-year-old fig trees (cv. “Dottato”) with excellent vegetative, productive, and phytosanitary conditions (Figure S1). After collection, the woody stems were temporarily stored in a cold room at 3 °C and 90% relative humidity. In February, the woody stems were prepared to obtain different cutting types [tip (T1), middle (T2), and basal portion (T3) of a one-year-old branch, as well as a two-year-old branch (T4)] and lengths [20 cm with 3–4 nodes (A) and 10 cm with 2–3 nodes (B)] (Figure S1). The bottom cut was made just below a node, while the upper cut was slanted 1–2 cm above the upper node. The prepared cuttings were immersed in a 0.2% fungicide solution (Dithane^®^ with active ingredient mancozeb 750 g/kg) to protect the cuttings from fungal infection.

### 2.2. Cutting, Rooting, and Evaluation of the Rooting Percentage

The rooting of cuttings was carried out in a greenhouse under a mist system. In particular, the cuttings were placed on basal-heated benches filled with perlite maintained at a constant temperature of 23 ± 2 °C (Figure S1). The bench bottom was heated at 24 °C, with the air temperature maintained at 20 ± 2 °C and the relative humidity at 60–70%. The cuttings were planted at a distance of 5 cm away from each other. The experimental setup followed a completely randomized block design in a factorial arrangement, with cutting types and length as the main factors. For each treatment, blocks of 18 cuttings with 8 replications for a total of 144 cuttings were provided (Figure S1). The cuttings were irrigated with the mist system every hour for 10 s to maintain favorable rooting conditions. The rooting rate was evaluated 30 days after planting. The percentage of rooting was calculated as the ratio of the number of rooted cuttings to the number of total cuttings × 100.

### 2.3. Transplanting and Growing Rooted Cuttings in Pots

After rooting evaluation, six rooted cuttings from each cutting type and length were transplanted into 0.60 L polyethylene pots filled with either perlite or a soil/peat/sand mixture (1:1:1, *v*:*v*:*v*). The perlite (Agrilit 2, Perlite Italiana Srl Corsico, Milano, Italy) had the following characteristics: 90% by volume of particles in the range of 1 to 3 mm, pile density of 60–80 kg/m^3^ ± 20%, and pH of 6.5–7.5. The peat used in the experiment was a blond sphagnum peat from Lithuanian peat bogs (Pac Italy srl Caldogno, Vicenza, Italy) with an acidic pH (4.5) and containing 40% organic carbon (C) of biological origin on dry matter, 0.5% organic nitrogen (N) on dry matter and 80% organic matter on dry matter. The sand came from a sand pit built for the requalification of the floodplain areas of the Crati River (Cosenza, south Italy). It was silica sand, which was washed, cycloned, sifted, and selected, with a grain size of 1 to 3 mm and free of weeds. The characteristics of the soil are provided in Table S1. Overall, a total of 48 rooted cuttings (4 cutting types × 2 cutting lengths × 6 replicates) for each growing medium were evaluated. These rooted cuttings were first acclimatized in an air-conditioned greenhouse (60–70% relative humidity and temperature of 20 ± 2 °C) for 20 days, with aerial irrigation provided every four hours for two minutes.

Cuttings in perlite were later transferred to another air-conditioned greenhouse (50–60% relative humidity; 22 ± 2 °C) and arranged in rows (10 cm intra-row and 100 cm inter-row spacing) as a randomized complete block design with six blocks for each treatment (Figure S1). Fertilization was provided by a drip irrigation system that included one emitter per pot and a flow rate of 2 L h^−1^. The following nutrient solution (mg L^−1^) was used: N (224), P (62), K (235), Ca (160), Mg (24), Fe (3), Mn (0.11), Cu (0.03), Zn (0.13), B (0.27), and Mo (0.05). The pH was kept in the range of 6.5 to 6.8 and EC was kept between 2.6–2.8 dS m^−1^. Fertigation was applied 3–4 times daily, providing 0.4 L per plant per day.

Cuttings in the soil/peat/sand mixture were transplanted into larger pots (30 cm height × 20 cm diameter) filled with the same mixture and placed in a shade house (35% shade) with an air temperature between 25 °C and 30 °C and relative humidity between 50–60%. A nebulization system was activated when the air temperature exceeded the threshold of 30 °C and the relative humidity dropped below 50%. These plants were arranged with 30 cm intra-row and 100 cm inter-row spacing, as a randomized complete block design with six blocks for each treatment. Pots were irrigated by a drip irrigation system with one emitter per pot and a flow rate of 4 L h^−1^. Pots were irrigated 3–4 times daily, depending on the leaf surface of self-rooted plants and the climate. Mineral nutrition was ensured by twice weekly fertigation with the following nutrient solution (mg L^−1^): N (130), P (11), K (42), Ca (36), Mg (6), Fe (3), Mn (0.1), Cu (0.03), Zn (0.4), B (0.05), and Mo (0.02). The EC values were kept within the range of 1.8–2.0 dS m^−1^, while the pH of the nutrient solution was maintained between 5.8 and 6.3. The amount of nutrient solution supplied to each plant for each fertigation was linked to plant development stages and it varied from a minimum of 0.5 L plant^−1^ (in the early stages of growth) to a maximum of 1 L plant^−1^ (in the final stages of growth).

### 2.4. Plant Biomass, Partitioning, and Above-Ground Morphology

First, 60 and 240 days after transplanting, six plants from each treatment (cutting type and length) and growing medium were collected and separated by leaf, shoot, cutting axes, and root system, which were used for morphological analyses. For each plant, the following measurements were recorded: shoot length (cm), the number of nodes (n), the number of leaves (n), fresh and dry stem weight (g), fresh and dry leaf weight (g), leaf area (cm^2^), fresh and dry cutting (part of the initial cutting) weight (g), and fresh and dry root system weight (g). The dry weights were measured after drying samples in a heated oven at 80 °C for the time required to obtain a constant weight. We also quantified the biomass allocation patterns to leaves, stems, trunks, and roots (% of the plant biomass). Total leaf area was measured using a LI-3100 Area meter, Li-Cor Biosciences, Lincoln, NE, USA. The average leaf area was obtained using the total leaf area/leaf number ratio.

### 2.5. Root Morphology Analysis

As above, the root systems were carefully washed to remove the substrate and subsequently stained with 0.1% toluidine blue solution for 5 min. Then, they were scanned at a resolution of 600 dpi (WinRhizo STD 1600, Instruments Régent Inc., Quebec, QC, Canada). To measure the following parameters, the WinRhizo Pro v. 4.0 software package (Instruments Régent Inc., Chemin Sainte-Foy, QC, Canada) was used: total length of the whole root system (RL, cm), total surface area of the whole root system (RSA, cm^2^), average diameter of the whole root system (RD, cm), and volume of the whole root system (RV, cm^3^). Moreover, the distribution of root length among the following root class diameters was measured [30]: very fine (vFi, 0–0.5 mm), fine (F, 0.5–1 mm), and coarse (C, >1 mm). The numbers of adventitious roots (NAr, n.) were directly counted from the images of the whole root system and the average length of the adventitious roots (aLAr, adventitious root axes plus lateral roots) was calculated. Afterwards, the fresh weights (RFW, g) and then the dry weights of the whole root system (RDW, g) were measured after oven-drying at 70 °C for 48 h. As reported by Ryser and Lambers [31], the following ‘components’ of the root length were calculated: root length ratio (RLR, total length of whole root system/whole plant dry weight, cm g^−1^), root mass ratio (RMR, dry weight of the whole root system/whole plant dry weight, g g^−1^), root fineness (RF, total length of the whole root system/volume of the whole root system, cm cm^−3^), and root tissue density (RTD, dry weight of the whole root system/volume of the whole root system, g cm^−3^).

### 2.6. Statistics

For statistical analysis, SPSS Statistics v. 15.0 software (IBM Corp., Armonk, NY, USA) was used. Graphics were prepared using SigmaPlot v. 8.0 software (Jandel Scientific, San Rafael, CA, USA). All data have been tested for normality (Kolmogorov Smirnoff test) and homogeneity of variance (Levene Median test) and, where required, the data have been transformed. Two-way ANOVA was performed to test the effects of the cutting types (CT) and length (CL), as well as the CT × CL interaction, on rooting percentage, plant biomass, and above-ground and root morphological parameters. Post hoc mean comparisons were conducted using Tukey’s test (*p* < 0.05). In order to capture the most relevant plant and root traits, a multivariate statistical approach was also used. In particular, two correlation matrixes constituted by the above-ground and root traits dataset (a total of 35 variables) obtained in the perlite and soil/peat/sand growing mediums, respectively, have been subjected to principal components analysis (PCA) [32]. The PCA produced uncorrelated multivariate axes that might be interpreted as representing a given fig cutting model in response to the experimental factors analyzed. The use of the correlation matrix standardizes differences among variables due to the measurement scale. The importance of different above-ground and root traits in a given axis is indicated by the relative loading of the traits in the eigenvector. Finally, correlations of biometric and above-ground and root morphological parameters obtained in the presence of perlite with those observed in the presence of the soil/peat/sand mixture were also evaluated using the Pearson test (significance at *p* < 0.05).

## 3. Result and Discussion

### 3.1. Cutting Rooting Ability

The rooting rate of cuttings from the fig cultivar Dottato ranged between 50% and 86.8% (Table 1), indicating a high ability to emit adventitious roots without hormone treatments when compared to other fig cultivars. Indeed, rooting percentages of 36%, 37%, 57%, 25–75%, 15–65%, and 35–44% have been measured for cv. Roxo de Valinhos [33], cv. Dinkar [34], cv. Brown [35], the cultivars Dane-Sefid, Vil, Pouz-Donbali, and Khormaei [18], the cultivars Bayoudhi, Jemaaoui, Ragoubi, Zidi, Bither, and Bouharrag [19], and cv. Roxo de Valinhos [36], respectively. However, similar rooting percentages to those in this study have been observed in different fig cultivars treated with hormones [37,38,39,40]. The cutting type and length significantly affected the rooting percentages of the Dottato cultivar, although no interaction effect has been observed (Table 1).

In particular, for cutting type, T4 (segment of a two-year-old branch) showed a significant increase of 53% and 25% compared to T1 (tip portion of a one-year-old branch) and T2 (middle portion of a one-year-old branch), respectively (Table 1). These findings indirectly confirmed that resource availability, mainly soluble sugar and starch levels in woody tissues, plays a critical role in root formation on cuttings through well-orchestrated hormone-related pathways and complex metabolic responses [41]. Notably, hardwood fig cuttings showed higher rooting rates than softwood ones [17,42]. Given the importance of cutting size (length and diameter) for the formation of adventitious roots [41], the scarcity of information on the role of cutting length in fig cultivars is surprising. Furthermore, it is crucial to determine the optimal cutting length in fig cultivars because (1) a longer cutting may waste valuable fig material, with limited or no added benefits to rooting percentage, while (2) shorter cuttings may reduce rooting success through insufficient storage reserves, leading to the very scarce development of the root system. In this study, longer cuttings resulted in a significantly higher rooting percentage in the Dottato cultivar than shorter ones (Table 1). Unexpectedly, this result was a bit different from that obtained by Aljane and Nahdi [19], who observed no effect of cutting length on the rooting percentage of the fig cultivar Bither. However, studies on other species indicated that intermediate cutting lengths were often suitable for propagation [43,44]. Nevertheless, most studies demonstrated that longer cuttings produced higher rooting percentages, such as in the blackberry [45], Eastern cottonwood [46], coffee [47], pear cultivars [48], and common osier [49].

#### 3.1.1. Biometric and Above-Ground Morphological Traits

The biometric traits and the biomass distribution within the plants of the Dottato fig cultivar, grown in perlite medium, were significantly changed by both cutting type and length, particularly affecting the above-ground biomass rather than the below-ground biomass (Table 2 and Table S2). Indeed, the fresh and dry biomass of the plant, shoot, leaf, and cutting, but not the root biomass, increased with the degree of cutting lignification: an increase of 2–3 times in the biomass was highlighted between the T1 and T4 cutting types (Table 2). In addition, the biomass was mostly addressed to the shoot system in T4 compared to the T1 cutting (Table S2). Cutting length modified the biomass traits in a similar way, as longer cuttings produced more biomass (Table 2). These results were expected because other authors have observed them in other fig cultivars [17,19,42,50], as well as related species such as Ficus Hawaii [16]. Physiologically, there is limited information on the molecular mechanisms by which different cutting types and lengths affect the above-ground system, especially in fig plants. However, it is speculated that fig plants, being characterized by strong apical dominancy, may exhibit reduced development when cuttings include apical (tip) buds, as opposed to buds located in the middle or basal portions. Additionally, resource availability (soluble sugar and starch levels in woody tissues) could be behind the higher biomass observed in T4 and the longer cuttings of cv. Dottato. Similar patterns have also been observed in cv. Dottato plants grown in a different medium (soil/peat/sand mixture) and cultivation system (Table 3 and Table S3). This finding confirmed that intrinsic factors of the cuttings determined the different fig developments among the diverse cutting types and lengths. In contrast to the biomass data, water content in the above-ground system was higher in less lignified (T1) and shorter cuttings in both different growth mediums and cultivation systems (Tables S2 and S3). This may be attributed to anatomical and structural differences in leaves and shoots among the fig plants from diverse cutting types and lengths. Indeed, leaf density, the proportion of leaf volume occupied by the mesophyll and epidermis, the mean size of cells and the thickness of cell walls, and the leaf mass area and its components affected the leaf’s water content [51]. The above-ground morphology exhibited similar trends to biometric traits across different growth media, with T4 and longer cuttings showing superior morphological characteristics (Tables S4 and S5).

#### 3.1.2. Root Morphological Traits

Table 4, Table 5, Table 6 and Table 7 report the results of the root system morphology of plants from the Dottato cultivar. Caruso’s studies [26,27] are the only works that describe, in detail, the fig root system at geometric, structural, and functional levels, albeit in response to AMF formulation. This study, however, is the first to investigate the detailed root morphology of fig plants as affected by different cutting types and lengths, grown in two distinct growth mediums and cultivation systems. Table 4 points out the root geometric parameters of the Dottato fig cultivar. In particular, the total length of the whole root system was statistically affected by the cutting type but not by cutting length in plants grown in perlite: segments from the 2-year-old branch (T4) showed a root system (11,815 cm) approximately 2- and 4-fold longer than that of T3 and T2 (5291 and 4769 cm) and T1 (3787 cm), respectively (Table 4). In contrast, the surface area of the whole root system was only modified by the cutting length: the surface area is significantly greater in longer cuttings (678 cm^2^) than in shorter ones (450 cm^2^) (Table 4).

Similar results were also found in cuttings grown in a soil/sand mixture (Table 5). In particular, the longer cuttings produced extensive root systems (127,220 cm) but, unlike with the perlite, the cutting type did not significantly affect root length. However, it is notable that T3 and T2 cuttings exhibited slightly greater lengths compared to T1 cuttings (Table 5). There are very little data in the literature investigating the length or surface area of the whole root system of different fig cutting types that, either directly or indirectly, support our results. For instance, Sivaji et al. [52] and Patel and Patel [53] showed that cutting types originating, respectively, from the basal trait and hardwood were characterized by a greater root system length. Aljane and Nahdi [19] observed that long cuttings (>60 cm) from a 1-year-old branch showed optimum rooting ability.

The geometric parameters of the root systems, such as length and surface area, are widely recognized as important soil exploration ‘indexes’ of the plant and, therefore, important for the acquisition of soil resources (water and nutrients) [54]. Therefore, plants with long and extensive root systems absorb more nutrients and water than other plants. While this assumption has been primarily tested in annuals and monocots such as grains [55] and rice [56], woody plants, including figs, also exhibit unique root characteristics, such as suberized root zones, which play a critical role in nutrient uptake, including nitrogen [57]. However, the root system length or surface area is insufficient to define the efficiency of the root system in acquiring soil resources. Other parameters, such as the length of the individual root types (in our case, only adventitious) and the root number, are crucial for plant survival and competitiveness in the soil. For example, lateral roots in wheat were observed to increase nitrate uptake [58], and second-order roots are important for water absorption in citrus species [25]. In addition, root tips were the preferential zones for nitrate uptake in citrus species [20]. In our study, the average length of the adventitious root was affected by the cutting type in fig plants grown in perlite, while the root number was modified by cutting length (Table 4). In fact, the T4 cutting displayed an adventitious root that was four times longer than other cutting types (480 cm vs. 181, 137, and 119 cm, Table 4), and longer cuttings generated a greater number of adventitious roots (36.9 vs. 27.6, Table 4). Although these effects were not statistically significant in the soil/sand mixture, a similar trend was observed: T4 cuttings and longer cuttings showed, respectively, a greater average length and a greater number of adventitious roots (Table 5). The increase in root emission in cuttings (during plant growth), as well as being important for nutrient and water absorption (greater root branching and tip zones), contributes favorably to transplant operations by minimizing root loss and enhancing the subsequent development of the root system [59]. Physiologically, the root elongation process, as well as the rooting process, depends on diverse factors such as the use of biomass and tensile stresses in the cell wall, which is governed by hormones and hormone-related genes, mainly auxins [60]. In this study, it is supposed that longer and basal fig cuttings could contain higher levels of carbohydrate reserves and auxins, facilitating rooting and root elongation processes [61]. Root diameter, a geometric parameter positively correlated with the construction cost of the root axes [62], is also modified by abiotic conditions in the soil. Many plants often reduce their root diameter in low-fertility soils [63] and very compact soils [64]. In our experiment, the average diameter of the fig root system was only marginally affected by cutting type and length in both growth mediums, although a significant interaction between CT and CL was observed in the soil/sand mixture (Table 4 and Table 5). So far, only considering the geometric parameters of the root (length, surface area, and average diameter) is insufficient to identify the “root ideotype”, a root system that is well-adapted to specific pedoclimatic environments. In fact, several authors have pointed out an array of traits or root phenes, singularly or synergistically, which offer additional insight into root system efficiency for adaptation to stressful environmental conditions [65]. Among these root traits, structural ones such as the root length ratio, the root mass ratio, root fineness, and root tissue density have been used for providing conclusions on the cost/benefit of the root system and, consequently, on the root efficiency [66]. For example, the root length ratio (cm of root per unit of whole plant dry weight) indicates “how much of the plant’s biomass is transformed into root length” and provides a better estimate of soil resource acquisition capacity under stress than absolute root length [66,67].

In this study, the RLR of the fig cuttings was significantly modified by cutting type in plants grown in perlite, with the T4 cutting showing a higher RLR (660 cm g^−1^) than other cutting types (Table 6). The RLR is a complex trait/phene that depends on several components, defined as “root length components”. These include the “allocation” component, which is the ratio of root mass (g of root dry weight per unit of dry weight of the whole plant), and the “structural” components, consisting of root fineness (root cm per unit root volume) and root tissue density (g root dry weight per unit root volume) [66]. Therefore, the plants can produce a long root system (high RLR) by increasing biomass allocation to the roots, increasing fineness, and/or reducing tissue density. For example, plants under water stress maintained a certain root length by reducing root tissue density [67] or by increasing root fineness [68]. Some authors have also observed a strong positive correlation between root fineness and water acquisition from the soil [25,69]. Therefore, in order to provide a complete and functional framework of the root system of fig plants, it was necessary to evaluate the RLR and its components (RMR, fineness, and tissue density) simultaneously.

Conversely to the RMR and tissue density, the fineness of the root system of fig plants grown in perlite was significantly changed by cutting type. The T4 cutting showed a root system three times finer than the other cutting types (3276 vs. 1022, 1173, and 1063 cm cm^−3^, Table 6). In the soil/sand mixture, root fineness was modified by cutting length with longer cuttings, resulting in finer roots (1450 vs. 1190 cm cm^−3^, Table 7). These results suggested that fig plants originating from the distal sections of two-year-old branches (T4) and with lengths of 20 cm were characterized by a high RLR due to increased root fineness.

The functional traits of roots are related to water and nutrient uptake in the root or transport efficiency within the root axes, as well as to mycorrhiza colonization. It is well-established that the fine and coarse roots exhibited absorptive and transport functions, respectively, and that they are defined by a diameter threshold that is <0.5 mm for very fine roots, 0.5–1 mm for fine roots, and >1 mm for coarse roots [30]. In perlite, the length of very fine roots was significantly modified by the cutting type. Once again, the T4 cuttings were characterized by an increase in the length of vFi roots relative to other cutting types (11,198 cm vs. 4656, 4231, and 3228 cm, Table 6). Conversely, in the soil/sand mixture, cutting length significantly affected the length of both vFi and Fi roots, with longer cuttings exhibiting higher values (113,842 and 12,618 cm vs. 78,328 and 10,893 cm, Table 7).

Therefore, these findings confirm that longer cuttings and T4 cuttings exhibited root systems mostly constituted by fine roots, which is consistent with the data on root fineness (Table 6 and Table 7). The large investment made by plants for the construction of fine roots has its ecological significance, especially for woody plants. In fact, fine roots are very responsive to changes in climatic conditions, inexpensive to construct, and, above all, they are root zones with a high capacity to absorb nutrients and water [69,70].

### 3.2. Multivariate Analysis

The univariate analysis used so far as a statistical test permitted us to identify the individual root or above-ground parameters of fig plants that were modified by cutting type and length. However, it is unable to identify “a set of traits” that could be used as proxies for fig cutting development and production and/or fitness when addressing climate change challenges. It is well-known, for instance, that individual root traits can cooperate synergistically for plant adaptation to variations in pedo-climatic conditions such as nutritional stress [65], water stress [71], and aluminum toxicity [72], as shown by various studies [14,73,74]. To this end, we applied multivariate analysis, specifically principal component analysis, to reduce and group all the above- and below-ground parameters of fig cuttings into ‘principal components’ that describe the variability between treatments (cutting type and length).

Table S6 shows the extracted fig cutting parameters (eight parameters) that were grouped into three principal components, explaining 88% of the variability among treatments in cuttings grown in perlite: 33%, 32%, and 23% for PC1, PC2, and PC3, respectively.

Further, Table S6 shows the factor loadings of each parameter within the PCs. In particular, it was observed that PC1 is characterized by high and positive factor loadings for leaf fresh biomass (0.879), with an average of 0.873, and total leaf area (0.974). Thus, PC1 could be biologically represented as “shoot morphology”. Positive and negative values for this component suggest an efficient and inefficient above-ground system, respectively, for resource acquisition in shoots (mainly light and CO_2_). PC2 showed high and positive factor loadings for root fresh (0.831) and dry biomass (0.909) and for the RMR (0.947) (Table S6). Considering that these parameters reflect resource allocation to the roots, we could define PC2 as the “root construction cost”. Positive values indicate greater use of resources for root construction at the expense of the plant’s reproductive and productive zones. Finally, PC3 was characterized by high and positive factor loadings for average root diameter (0.899) and root fineness (0.858) (Table S6). Thus, this component could discriminate by high or low “root fineness”, suggesting a higher or poor root capacity for soil resource acquisition.

**Figure 1:**
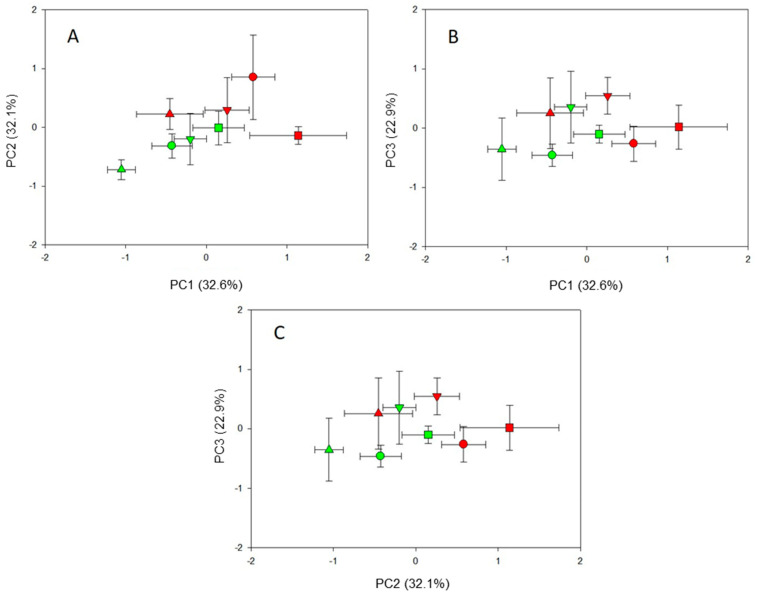
Biplot of the scores (means and standard error bars) of PC1 vs. PC2 (**A**), PC1 vs. PC3 (**B**), and PC2 vs. PC3 (**C**) that grouped the biometric and morphological parameters within the fig plants (cv. Dottato) of different cutting types (T1: tip portion of a one-year-old branch; T2: middle portion of a one-year-old branch; T3: basal portion of a one-year-old branch; T4: segment of a two-year-old branch) and lengths (A: 20 cm with 2–3 nodes; B: 10 cm with 3–4 nodes) 60 days after transplantation into a pot filled with perlite. The significant morpho-physiological and yield parameters are reported in Table S6. The proportion of explained variability is given within the bracket. Scores related to the cutting type and length are indicated by different symbols and colors, respectively: T1A (▲), T1B (▲), T2A (●), T2B (●), T3A (■), T3B (■), T4A (▼), T4B (▼).

Figure 1 shows the biplot of the PCs.

In particular, the PC1 vs. PC2 biplot (Figure 1A) highlighted that (i) PC1 separates cutting types, showing that apical cuttings (T1) exhibited less efficient shoot morphology compared to distal segments (T3 and T4); (ii) PC2 separates cutting lengths, indicating that longer cuttings (primarily T1 and T2) were associated with higher root construction costs; and (iii) cuttings originating from the basal portion of a one-year-old branch (T3) and measuring 20 cm in length demonstrated optimal shoot morphology with lower root construction costs. The PC1 vs. PC3 biplot revealed that longer cuttings from two-year-old branches (T4) and basal portion of one-year-old branches (T3) exhibited optimal shoot morphology with high root fineness (Figure 1B). In contrast, shorter cuttings from the apical and middle portions of one-year-old branches (T1 and T2) displayed the opposite trend (Figure 1C). Finally, the PC2 vs. PC3 biplot demonstrated that longer cuttings, particularly T4 and T3, were associated with high root construction costs, which corresponded with increased root fineness (Figure 1C). Conversely, shorter cuttings (T1 and T2) showed a completely opposite pattern (Figure 1C).

**Figure 2:**
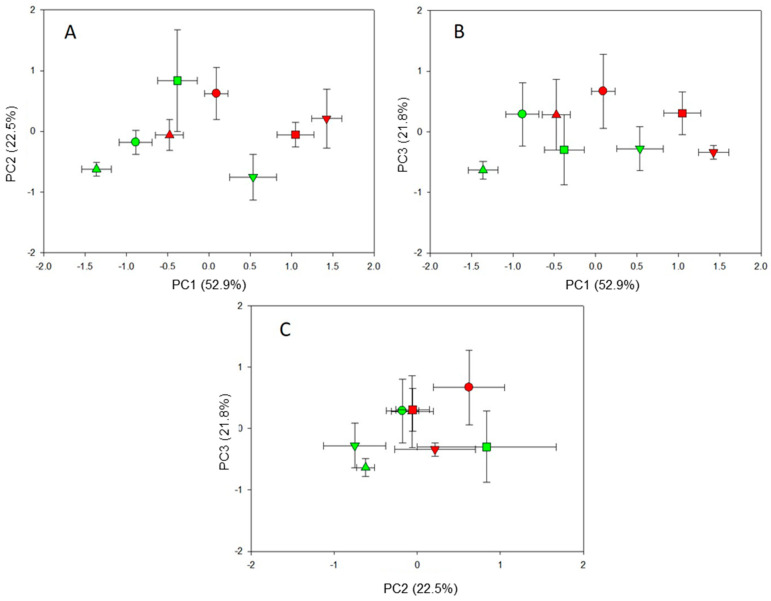
Biplot of the scores (means and standard error bars) of PC1 vs. PC2 (**A**), PC1 vs. PC3 (**B**), and PC2 vs. PC3 (**C**) that grouped the biometric and morphological parameters of the fig plants (cv. Dottato) of different cutting types (T1: tip portion of a one-year-old branch; T2: middle portion of a one-year-old branch; T3: basal portion of a one-year-old branch; T4: segment of a two-year-old branch) and lengths (A: 20 cm with 2–3 nodes; B: 10 cm with 3–4 nodes) 240 days after transplantation into a pot filled with a soil/peat/sand mixture. The significant morpho-physiological and yield parameters are reported in Table S7. The proportion of explained variability is given within the bracket. Scores related to the cutting type and length are indicated by different symbols and colors, respectively: T1A (▲), T1B (▲), T2A (●), T2B (●), T3A (■), T3B (■), T4A (▼), T4B (▼).

Table S7 and Figure 2 show the results of the principal components analysis for fig cuttings grown in a soil/sand mixture. The test has yielded three principal components with, respectively, 52.9%, 22.5%, and 21.8% of the variability among treatments for a total of 97%.

Table S7 reports the identified parameters and how they are distributed into three principal components. In particular, PC1 is defined by high and positive factor loadings for shoot fresh and dry biomass, leaf fresh and dry biomass, shoot length, leaf number, and surface area. Obviously, this component represents ‘shoot morphology’, where positive loading values indicate an optimal shoot shape for resource acquisition. PC2 consisted of high and positive loadings for the total length of the whole root system, RLR, and length of very fine roots (Table S7). Positive coefficients of this principal component indicated a very long and fine root system and, thus, this component is defined as “root geometry and function”. Finally, PC3 includes high and positive loading for root fresh and dry biomass and the RMR (Table S7), representing the “root construction cost”. The biplots for these components reveal trends similar to those observed in perlite-grown cuttings. Plants originating from the distal segments of 1-year-old branches (T2, T3, and T4 cuttings), especially long cuttings, had (1) an efficient shoot morphology and root system optimized for soil resource acquisition (long and very fine roots) (Figure 2A) and (2) exhibited lower and higher root construction costs for the T4 and T3 cuttings, respectively (Figure 2B,C). The fig plants originating from T1 cuttings and shorter cuttings exhibited an opposite pattern (Figure 2).

Overall, from two different experiments, the PCA results suggested that distal cutting segments (T3 and T4), especially those with a long size (20 cm), produced fig plants characterized by well-structured and efficient above- and below-ground organs for acquiring atmospheric (light and CO_2_) and soil (water and nutrients) resources with a lower construction cost.

### 3.3. Correlations Between Cutting Growth and Morphology in the Soil/Peat/Sand Mixture and in Perlite

**Figure 3:**
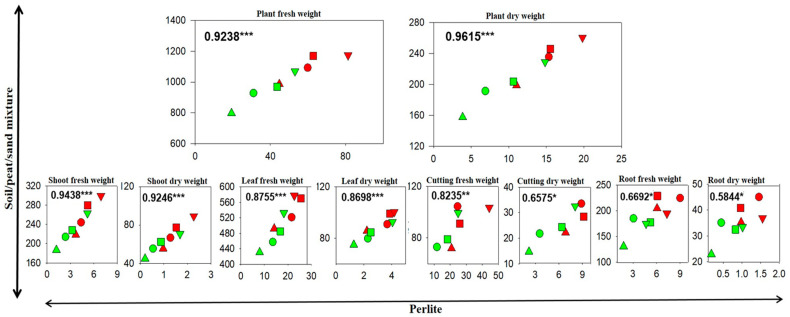
Correlations between the biometric parameters of the fig plants (cv. Dottato) from different cutting types and lengths 60 days after transplantation into a pot filled with perlite and 240 days after transplantation into a pot filled with a soil/peat/sand mixture. The cutting type (T1: tip portion of a one-year-old branch; T2: middle portion of a one-year-old branch; T3: basal portion of a one-year-old branch; T4: segment of a two-year-old branch) and length (A: 20 cm with 2–3 nodes; B: 10 cm with 3–4 nodes) are indicated by different symbols and colors, respectively: T1A (▲), T1B (▲), T2A (●), T2B (●), T3A (■), T3B (■), T4A (▼), T4B (▼). The numbers within the figures indicate the coefficient of determination, while the statistic (Pearson test) is described as follows: * 0.05 > *p* > 0.01; ** 0.01 > *p* > 0.001; *** 0.001 > *p*.

**Figure 4:**
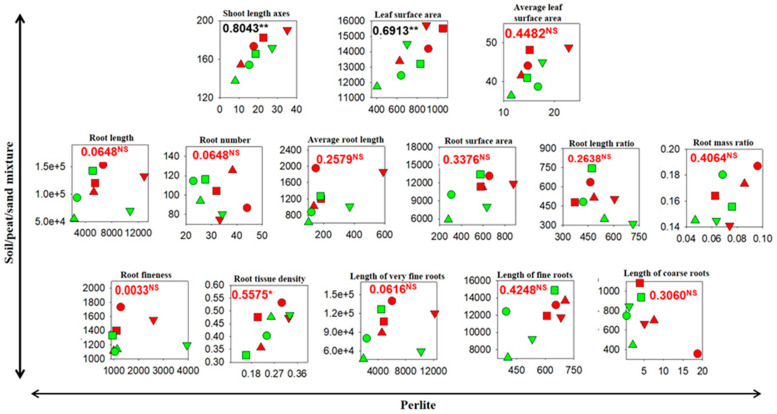
Correlations between the morphological parameters of the fig (cv. Dottato) plants from different cutting types and lengths 60 days after transplantation into a pot filled with perlite and 240 days after transplantation into a pot filled with a soil/peat/sand mixture. The cutting type (T1: tip portion of a one-year-old branch; T2: middle portion of a one-year-old branch; T3: basal portion of a one-year-old branch; T4: segment of a two-year-old branch) and length (A: 20 cm with 2–3 nodes; B: 10 cm with 3–4 nodes) are indicated by different symbols and colors, respectively: T1A (▲), T1B (▲), T2A (●), T2B (●), T3A (■), T3B (■), T4A (▼), T4B (▼). The numbers within the figures indicate the coefficient of determination, while the statistic (Pearson test) is described as follows: * 0.05 > *p* > 0.01; ** 0.01 > *p* > 0.001; ^NS^—not significant.

Unlike soil, soilless mediums such as perlite are widely used in crop research because they permit (1) the fine control of treatments and environmental variables, resulting in more consistent and highly reproducible results, (2) the use of large sample numbers, (3) a faster screening process and, finally, (4) the study of the root’s form and function. While perlite or other inert materials or hydroponics are not soil substitutes in toto, several studies have reported comparable results between hydroponic and field systems [75,76]. Knowing that perlite cannot be a complete substitute for soil and that findings obtained with it need to be validated through field experiments, we tested whether the biometric and morphological patterns of rooted cuttings obtained in perlite are comparable to those from the soil/peat/sand mixture. Figure 3 and Figure 4 show the correlation results, pointing out statistically significant relationships between the biometric and morphological parameters of the shoot (*p* < 0.001) and the biometric parameters of the root (0.05 < *p* < 0.01), unlike those of the root morphology. Most likely, the lower or non-existent statistical significance for root biometric and morphology traits may be caused by different factors, such as the loss of roots during soil extraction and image acquisition and the different physic-chemical conditions of the soil between the two growth mediums.

## 4. Conclusions

In conclusion, this study pointed out the significant effects of cutting type, length, and growth medium on the rooting ability, biomass production, and root morphology of the fig cultivar Dottato. The findings demonstrate that distal (T3 and T4) and longer cuttings, particularly those from two-year-old branches, exhibited superior rooting success, increased biomass, and extensive root development when compared to shorter and apical cuttings (T1 and T2). These characteristics should be considered for improving and optimizing nursery production and ensuring transplant success, as they contribute to both above- and below-ground resilience. The multivariate analysis further emphasizes the importance of specific root traits, such as root fineness and length, in adapting to diverse growth environments, suggesting that targeted cutting selection, mainly distal and longer cuttings, can optimize nursery propagation and support the species’ adaptation to varying environmental conditions such as those resulting from climate change. Future research is recommended to validate these findings under field conditions, exploring further potential for enhancing fig cultivation in diverse pedo-climatic settings.

## Figures and Tables

**Table 1:** Rooting rate (%) (mean ± standard error) of the different cutting types (T1: tip portion of a one-year-old branch; T2: middle portion of a one-year-old branch; T3: basal portion of a one-year-old branch; T4: segment of a two-year-old branch) and length (A: 20 cm with 2–3 nodes; B: 10 cm with 3–4 nodes) of fig cuttings (cv. Dottato) after 30 days after their planting in a heated bed.

Parameter	Statistics ^#^	Cutting Length (CL)	Cutting Type (CT)				CL Average
			T1	T2	T3	T4	
Rooting rate (%)	CT 19.26 *** CL 11.36 ** CT × CL 0.56 ^NS^	**A**	59.0 ± 1.8	76.4 ± 3.9	81.3 ± 5.0	86.8 ± 3.1	75.9 ^X^
		**B**	50.0 ± 3.3	60.4 ± 4.7	73.6 ± 3.1	80.6 ± 4.2	66.2 ^Y^
		**CT average**	54.5 ^C^	68.4 ^B^	77.4 ^AB^	83.7 ^A^	

Capital letters indicate significant differences among the means along the rows (*p* < 0.05, Tukey’s test). The letters are reported only in the case of statistical significance of the individual factors and their interaction. ^#^ Statistics: two-way ANOVA with eight replicates (CT: cutting type; CL: cutting length; CT × CL: cutting type × cutting length interaction; the number indicates the F values, while the *p*-values are indicated as follows: ** 0.01 > *p* > 0.001; *** 0.001 > *p*; ^NS^—not significant).

**Table 2:** Fresh and dry biomass (average ± standard deviation) of the fig plant (cv. Dottato) and organs of different cutting types (T1: tip portion of a one-year-old branch; T2: middle portion of a one-year-old branch; T3: basal portion of a one-year-old branch; T4: segment of a two-year-old branch) and lengths (A: 10 cm with 2–3 nodes; B: 20 cm with 3–4 nodes) 60 days after transplantation in pots filled with perlite.

Parameters	Statistics ^#^	Cutting Length (CL)	Cutting Type (CT)				CL Average
			T1	T2	T3	T4	
Plant fresh biomass (g)	CT 18.55 *** CL 55.73 *** CT × CL 0.41 ^NS^	**A**	45.3 ^a^ ± 12.3	60.4 ^a^ ± 11.7	63.3 ^a^ ± 11.3	81.1 ^a^ ± 17.3	62.2 ^X^
		**B**	19.2 ^b^ ± 4.0	31.3 ^b^ ± 8.2	44.2 ^b^ ± 13.1	53.3 ^b^ ± 14.2	36.8 ^Y^
		**CT average**	32.3 ^C^	45.97 ^BC^	53.8 ^AB^	67.2 ^A^	
Shoot axes fresh biomass (g)	CT 12.63 *** CL 22.73 *** CT × CL 0.14 ^NS^	**A**	3.7 ^a^ ± 1.2	4.4 ^a^ ± 1.8	5.2 ^a^ ± 0.9	6.9 ^a^ ± 2.1	5.1 ^X^
		**B**	1.2 ^b^ ± 0.3	2.4 ^b^ ± 1.6	3.2 ^b^ ± 1.6	1.7 ^b^ ± 0.9	3.0 ^Y^
		**CT average**	2.45 ^C^	3.41 ^BC^	4.21 ^B^	6.04 ^A^	
Leaf fresh biomass (g)	CT 9.52 *** CL 21.00 *** CT × CL 0.43 ^NS^	**A**	14.2 ^a^ ± 7.0	21.7 ^a^ ± 4.5	26.0 ^a^ ± 6.0	23.0 ^a^ ± 5.0	21.2 ^X^
		**B**	8.0 ^b^ ± 2.6	13.7 ^b^ ± 4.8	17.0 ^b^ ± 6.0	18.0 ^b^ ± 4.0	14.2 ^Y^
		**CT average**	11.1 ^B^	17.7 ^AB^	21.5 ^A^	20.5 ^A^	
Cutting fresh biomass (g)	CT 21.57 *** CL 48.88 *** CT × CL 1.65 ^NS^	**A**	21.0 ^a^ ± 6.0	25.0 ^a^ ± 6.0	26.0 ^a^ ± 7.0	44.0 ^a^ ± 11.0	29.0 ^X^
		**B**	8.0 ^b^ ± 2.0	12.0 ^b^ ± 13.0	18.0 ^b^ ± 5.0	25.0 ^b^ ± 7.0	15.9 ^Y^
		**CT average**	14.6 ^B^	18.4 ^AB^	22.2 ^AB^	34.7 ^A^	
Root fresh biomass (g)	CT 1.60 ^NS^ CL 18.90 *** CT × CL 1.87 ^NS^	**A**	6.1 ^a^ ± 2.6	9.0 ^a^ ± 4.1	6.1 ^a^ ± 1.2	7.3 ^a^ ± 3.6	7.1
		**B**	1.8 ^b^ ± 0.5	3.1 ^b^ ± 1.2	5.2 ^b^ ± 2.6	4.7 ^b^ ± 3.2	3.7
		**CT average**	3.9 ^A^	6.1 ^A^	5.7 ^A^	6.0 ^A^	
Plant dry biomass (g)	CT 22.50 *** CL 54.07 *** CT × CL 1.00 ^NS^	**A**	11.0 ± 3.2	15.4 ± 3.3	15.5 ± 3.3	19.8 ± 3.9	15.4 ^X^
		**B**	3.9 ± 0.7	6.9 ± 1.4	10.6 ± 3.3	14.8 ± 3.5	9.1 ^Y^
		**CT average**	7.4 ^B^	11.1 ^B^	13.1 ^AB^	17.3 ^A^	
Shoot axes dry biomass (g)	CT 12.41 *** CL 16.64 ** CT × CL 0.06 ^NS^	**A**	1.0 ± 0.4	1.3 ± 0.5	1.5 ± 0.2	2.3 ± 1.1	1.5 ^X^
		**B**	0.2 ± 0.1	0.6 ± 0.3	0.9 ± 0.5	1.7 ± 0.9	0.8 ^Y^
		**CT average**	0.6 ^C^	0.9 ^B^	1.2 ^B^	1.2 ^A^	
Leaf dry biomass (g)	CT 12.04 *** CL 11.52 ** CT × CL 1.08 ^NS^	**A**	2.2 ± 0.4	3.7 ± 0.4	3.9 ± 0.5	4.2 ± 0.5	3.5 ^X^
		**B**	1.3 ± 0.2	2.3 ± 0.3	2.5 ± 0.3	4.1 ± 0.4	2.5 ^Y^
		**CT average**	1.8 ^B^	3.0 ^AB^	3.2 ^AB^	4.1 ^A^	
Cutting dry biomass (g)	CT 18.15 *** CL 58.12 *** CT × CL 1.05 ^NS^	**A**	6.9 ± 0.9	8.9 ± 1.1	9.2 ± 1.0	11.8 ± 0.9	9.2 ^X^
		**B**	2.2 ± 0.2	3.6 ± 0.3	6.4 ± 0.8	8.1 ± 0.6	5.1 ^Y^
		**CT average**	4.52 ^B^	6.25 ^AB^	7.79 ^AB^	9.94 ^A^	
Root dry biomass (g)	CT 1.89 ^NS^ CL 8.64 ** CT × CL 0.81 ^NS^	**A**	1.0 ± 0.2	1.5 ± 0.5	1.0 ± 0.1	1.6 ± 0.5	1.2 ^X^
		**B**	0.2 ± 0.1	0.5 ± 0.1	0.8 ± 0.2	1.0 ± 0.4	0.6 ^Y^
		**CT average**	0.6 ^A^	1.0 ^A^	0.9 ^A^	1.3 ^A^	

Lowercase letters indicate significant differences among the means within columns (*p* < 0.05. Tukey’s test). Capital letters indicate significant differences among the means along the rows (*p* < 0.05. Tukey’s test). The letters are only reported in the case of statistical significance of the individual factors and their interaction. ^#^ Statistics: two-way ANOVA with six replicates (CT: cutting type; CL: cutting length; CT × CL: cutting type × cutting length interaction; the number indicates the F values, while the *p*-values are indicated as follows: ** 0.01 > *p* > 0.001; *** 0.001 > *p*; ^NS^—not significant).

**Table 3:** Fresh and dry biomass (average and standard deviation) of the fig plant (cv. Dottato) and organs of different cutting types (T1: tip portion of a one-year-old branch; T2: middle portion of a one-year-old branch; T3: basal portion of a one-year-old branch; T4: segment of a two-year-old branch) and lengths (A: 10 cm with 2–3 nodes; B: 20 cm with 3–4 nodes) 240 days after transplantation in a pot filled with a soil/peat/sand mixture.

Parameters	Statistics ^#^	Cutting Length (CL)	Cutting Type (CT)				CL Average
			T1	T2	T3	T4	
Plant fresh biomass (g)	CT 13.63 *** CL 38.09 *** CT × CL 0.64 ^NS^	**A**	987 ± 26	1092 ± 40	1170 ± 48	1174 ± 31	1106 ^X^
		**B**	798 ± 26	927 ± 32	969 ± 52	1070 ± 37	941 ^Y^
		**CT average**	893 ^C^	1009 ^B^	1069 ^AB^	1122 ^A^	
Shoot axes fresh biomass (g)	CT 53.43 *** CL 66.07 *** CT × CL 0.14 ^NS^	**A**	219 ± 5	243 ± 2	280 ± 7	299 ± 7	260 ^X^
		**B**	187 ± 6	213 ± 6	228 ± 8	263 ± 9	223 ^Y^
		**CT average**	203 ^D^	228 ^C^	254 ^B^	281 ^A^	
Leaf fresh biomass (g)	CT 19.05 *** CL 45.47 *** CT × CL 0.76 ^NS^	**A**	492 ± 8	521 ± 7	570 ± 16	577 ± 12	540 ^X^
		**B**	431 ± 12	457 ± 12	485 ± 16	532 ± 18	476 ^Y^
		**CT average**	461 ^B^	489 ^B^	528 ^A^	555 ^A^	
Cutting fresh biomass (g)	CT 8.19 ** CL 8.71 ** CT × CL 1.01 ^NS^	**A**	72 ± 9	104 ± 8	91 ± 7	103 ± 12	93 ^X^
		**B**	50 ± 5	73 ± 5	79 ± 8	99 ± 10	75 ^Y^
		**CT average**	61 ^B^	88 ^A^	85 ^A^	101 ^A^	
Root fresh biomass (g)	CT 1.38 ^NS^ CL 9.76 ** CT × CL 0.59 ^NS^	**A**	204 ± 25	224 ± 33	229 ± 23	194 ± 5	213 ^X^
		**B**	130 ± 10	184 ± 20	177 ± 23	175 ± 15	167 ^Y^
		**CT average**	167	204	203	185	
Plant dry biomass (g)	CT 16.86 *** CL 33.78 *** CT × CL 0.17 ^NS^	**A**	199 ± 8	235 ± 11	246 ± 10	261 ± 11	235 ^X^
		**B**	158 ± 5	191 ± 9	204 ± 13	229 ± 9	195 ^Y^
		**CT average**	178 ^C^	213 ^B^	225 ^AB^	245 ^A^	
Shoot axes dry biomass (g)	CT 96.12 *** CL 115.11 *** CT × CL 2.06 ^NS^	**A**	55 ± 0.5	66 ± 1.5	77 ± 1.9	89 ± 2.1	72 ^X^
		**B**	45 ± 1.2	55 ± 2.0	62 ± 2.6	71 ± 1.9	58 ^Y^
		**CT average**	50 ^D^	61 ^C^	70 ^B^	80 ^A^	
Leaf dry biomass (g)	CT 19.05 *** CL 45.47 *** CT × CL 0.76 ^NS^	**A**	86 ± 1.4	91 ± 1.3	99 ± 2.9	101 ± 2.1	94 ^X^
		**B**	75 ± 2.1	80 ± 2.1	84 ± 2.8	93 ± 3.2	83 ^Y^
		**CT average**	80 ^B^	85 ^B^	92 ^A^	97 ^A^	
Cutting dry biomass (g)	CT 7.84 *** CL 8.06 ** CT × CL 0.98 ^NS^	**A**	22 ± 3	33 ± 3	28 ± 2	34 ± 5	30 ^X^
		**B**	15 ± 2	22 ± 2	24 ± 3	33 ± 4	23 ^Y^
		**CT average**	19 ^B^	28 ^A^	26 ^AB^	33 ^A^	
Root dry biomass (g)	CT 1.78 ^NS^ CL 6.22 * CT × CL 0.31 ^NS^	**A**	35 ± 6	45 ± 7	41 ± 4	37 ± 3	39 ^X^
		**B**	23 ± 1	35 ± 5	32 ± 5	33 ± 4	31 ^Y^
		**CT average**	29	40	37	35	

Capital letters indicate significant differences among the means along the rows (*p* < 0.05, Tukey’s test). The letters are only reported in the case of statistical significance of the individual factors and their interaction. ^#^ Statistics: two-way ANOVA with six replicates (CT: cutting type; CL: cutting length; CT × CL: cutting type × cutting length interaction; the number indicates the F values, while the *p*-values are indicated as follows: * 0.05 > *p* > 0.01; ** 0.01 > *p* > 0.001; *** 0.001 > *p*; ^NS^—not significant).

**Table 4:** Root geometry (average ± standard deviation) of the fig plants (cv. Dottato) of different cutting types (T1: tip portion of a one-year-old branch; T2: middle portion of a one-year-old branch; T3: basal portion of a one-year-old branch; T4: segment of a two-year-old branch) and lengths (A: 20 cm with 2–3 nodes; B: 10 cm with 3–4 nodes) 60 days after transplantation into a pot filled with perlite.

Parameters	Statistics ^#^	Cutting Length (CL)	Cutting Type (CT)				CL Average
			T1	T2	T3	T4	
Total length of the whole root system (cm)	CT 6.29 *** CL 2.59 ^NS^ CT × CL 0.2750 ^NS^	**A**	5273 ± 3048	6734 ± 3808	5458 ± 2523	12,883 ± 11,166	7587
		**B**	2302 ± 2001	2803 ± 1735	5125 ± 3105	10,746 ± 5663	5244
		**CT average**	3787 ^B^	4769 ^B^	5291 ^B^	11,815 ^A^	
Total surface area of the whole root system (cm^2^)	CT 2.17 ^NS^ CL 5.72 * CT × CL 0.67 ^NS^	**A**	596 ± 358	659 ± 213	579 ± 248	878 ± 541	678 ^x^
		**B**	281 ± 296	311 ± 190	575 ± 305	634 ± 353	450 ^y^
		**CT average**	439	485	577	756	
Average diameter of the whole root system (cm)	CT 1.60 ^NS^ CL 0.83 ^NS^ CT × CL 0.23 ^NS^	**A**	0.60 ± 0.59	0.43 ± 0.60	0.53 ± 0.36	0.73 ± 0.37	0.58
		**B**	0.38 ± 0.24	0.37 ± 0.18	0.44 ± 0.18	0.73 ± 0.64	0.48
		**CT average**	0.50	0.40	0.49	0.73	
Adventitous root number (n)	CT 0.23 ^NS^ CL 5.74 * CT × CL 1.57 ^NS^	**A**	38.33 ± 15.37	44.17 ± 15.37	32.00 ± 8.07	33.33 ± 20.06	36.9 ^x^
		**B**	25.67 ± 7.94	23.00 ± 11.52	27.67 ± 6.89	34.33 ± 16.13	27.6 ^y^
		**CT average**	32.00	33.58	29.83	33.83	
Average length of the adventitous root system (cm)	CT 4.00 ** CL 0.71 ^NS^ CT × CL 0.34 ^NS^	**A**	136 ± 66	151 ± 56	182 ± 97	590 ± 766	265
		**B**	102 ± 96	122 ± 73	180 ± 85	371 ± 254	194
		**CT average**	119 ^B^	137 ^B^	181 ^AB^	480 ^A^	

Lowercase letters indicate significant differences among the means within columns (*p* < 0.05, Tukey’s test). Capital letters indicate significant differences among the means along the rows (*p* < 0.05, Tukey’s test). The letters are only reported in the case of statistical significance of the individual factors and their interaction. ^#^ Statistics: two-way ANOVA with six replicates (CT: cutting type; CL: cutting length; CT × CL: cutting type × cutting length interaction; the number indicates the F values, while the *p*-values are indicated as follows: * 0.05 > *p* > 0.01; ** 0.01 > *p* > 0.001; *** 0.001 > *p*; ^NS^—not significant).

**Table 5:** Root geometry (average ± standard deviation) of the fig plants (cv. Dottato) of different cutting types (T1: tip portion of a one-year-old branch; T2: middle portion of a one-year-old branch; T3: basal portion of a one-year-old branch; T4: segment of a two-year-old branch) and lengths (A: 20 cm with 2–3 nodes; B: 10 cm with 3–4 nodes) 240 days after transplantation into a pot filled with a soil/peat/sand mixture.

Parameters	Statistics ^#^	Cutting Length (CL)	Cutting Type (CT)				CL Average
			T1	T2	T3	T4	
Total length of the whole root system (cm)	CT 2.17 ^NS^ CL 5.59 * CT × CL 1.64 ^NS^	**A**	103,766 ± 35,088	152,747 ± 67,185	119,838 ± 36,323	132,528 ± 63,805	127,220 ^x^
		**B**	55,295 ± 12,306	92,783 ± 27,501	142,410 ± 76,696	69,542 ± 43,411	90,007 ^y^
		**CT average**	79,530	122,765	131,124	101,035	
Total surface area of the whole root system (cm^2^)	CT 1.58 ^NS^ CL 3.56 ^NS^ CT × CL 1.40 ^NS^	**A**	11,261 ± 2995	13,131 ± 5624	11,380 ± 2968	11,859 ± 5358	11,908
		**B**	5856 ± 850	10,005 ± 2675	13,442 ± 4636	8021 ± 6464	9330
		**CT average**	8559	11,568	12,410	9940	
Average diameter of the whole root system (cm)	CT 0.84 ^NS^ CL 0.14 ^NS^ CT × CL 4.27 *	**A**	0.56 ^a^ ± 0.32	0.53 ^a^ ± 0.37	0.34 ^b^ ± 0.11	0.32 ^a^ ± 0.19	0.44
		**B**	0.27 ^b^ ± 0.10	0.29 ^b^ ± 0.07	0.69 ^a^ ± 0.20	0.39 ^a^ ± 0.27	0.41
		**CT average**	0.41	0.41	0.51	0.36	
Adventitous root number (n)	CT 1.82 ^NS^ CL 0.09 ^NS^ CT × CL 1.22 ^NS^	**A**	126 ± 73	86 ± 39	104 ± 19	75 ± 23	101
		**B**	94 ± 25	114 ± 34	116 ± 21	80 ± 20	98
		**CT average**	109.80	100.30	110.20	77.50	
Average length of the adventitous root system (cm)	CT 2.17 ^NS^ CL 5.59 * CT × CL 1.64 ^NS^	**A**	1019 ± 662	1948 ± 816	1199 ± 457	1866 ± 841	1508 ^x^
		**B**	624 ± 201	866 ± 292	1270 ± 680	1011 ± 776	942 ^y^
		**CT average**	822	1407	1235	1438	

Lowercase letters indicate significant differences among the means within columns (*p* < 0.05, Tukey’s test). The letters are only reported in the case of statistical significance of the individual factors and their interaction. ^#^ Statistics: two-way ANOVA with six replicates (CT: cutting type; CL: cutting length; CT × CL: cutting type × cutting length interaction; the number indicates the F values, while the *p*-values are indicated as follows: * 0.05 > *p* > 0.01; ^NS^—not significant).

**Table 6:** Root length components [RLR (cm root length/g plant dry biomass): root length ratio; RMR (g root dry biomass/g plant dry biomass): root mass ratio; root fineness (cm root length/cm^3^ root volume); and root tissue density (g root dry biomass/cm^3^ root volume)] and root functional traits as root length distribution (average and standard deviation) among the different diameter classes [vFi (very fine): <0.05 mm; Fi (fine): 0.05 < D < 1 mm; and C (coarse): >1 mm)] (average ± standard deviation) of the root system of fig plants (cv. Dottato) of different cutting types (T1: tip portion of a one-year-old branch; T2: middle portion of a one-year-old branch; T3: basal portion of a one-year-old branch; T4: segment of a two-year-old branch) and lengths (A: 20 cm with 2–3 nodes; B: 10 cm with 3–4 nodes) 60 days after transplantation into a pot filled with perlite.

Parameters	Statistics ^#^	Cutting Length (CL)	Cutting Type (CT)				CL Average
			T1	T2	T3	T4	
RLR (cm/g)	CT 1.43 * CL 0.40 ^NS^ CT × CL 0.15 ^NS^	**A**	484 ± 236	464 ± 264	378 ± 227	606 ± 443	48
		**B**	545 ± 385	422 ± 279	467 ± 240	714 ± 338	538
		**CT average**	515 ^AB^	443 ^AB^	422 ^B^	660 ^A^	
RMR (g/g)	CT 0.37 ^NS^ CL 1.75 ^NS^ CT × CL 0.89 ^NS^	**A**	0.09 ± 0.03	0.10 ± 0.08	0.05 ± 0.02	0.07 ± 0.05	0.08
		**B**	0.05 ± 0.01	0.07 ± 0.04	0.09 ± 0.05	0.06 ± 0.04	0.06
		**CT average**	0.07	0.08	0.07	0.07	
Root fineness (cm/cm^3^)	CT 22.62 *** CL 1.42 ^NS^ CT × CL 2.55 ^NS^	**A**	993 ± 171	1291 ± 881	1100 ± 169	2600 ± 1421	1496
		**B**	1132 ± 352	1055 ± 154	944 ± 209	3953 ± 1438	1771
		**CT average**	1063 ^B^	1173 ^B^	1022 ^B^	3276 ^A^	
Root tissue density (g/cm^3^)	CT 1.11 ^NS^ CL 0.07 ^NS^ CT × CL 0.16 ^NS^	**A**	0.22 ± 0.17	0.30 ± 0.26	0.17 ± 0.08	0.33 ± 0.19	0.26
		**B**	0.26 ± 0.37	0.24 ± 0.16	0.22 ± 0.17	0.33 ± 0.14	0.25
		**CT average**	0.24	0.27	0.183	0.33	
vFi (cm)	CT 6.69 *** CL 2.39 ^NS^ CT × CL 0.24 ^NS^	**A**	4560 ± 2625	6056 ± 3790	4840 ± 2325	12,190 ± 10,990	6912
		**B**	1896 ± 1490	2410 ± 1550	4470 ± 2950	10,210 ± 5320	4744
		**CT average**	3228 ^B^	4231 ^B^	4656 ^B^	11,198 ^A^	
Fi (cm)	CT 0.22 ^NS^ CL 2.77 ^NS^ CT × CL 0.58 ^NS^	**A**	704.20 ± 429	657.20 ± 176	608.60 ± 232	682 ± 364	663
		**B**	403.90 ± 570	396.50 ± 227	650.05 ± 174	531 ± 401	495
		**CT average**	554	526	629	606	
C (cm)	CT 0.95 ^NS^ CL 5.08 * CT × CL 1.77 ^NS^	**A**	7.60 ± 15.90	18.80 ± 21.90	3.90 ± 4.40	5.10 ± 6.20	8.86 ^X^
		**B**	2.20 ± 4.50	0.65 ± 0.80	4.30 ± 6.80	1.20 ± 3.00	2.10 ^Y^
		**CT average**	4.88	9.70	4.10	3.20	

Capital letters indicate significant differences among the means along the rows (*p* < 0.05, Tukey’s test). The letters are only reported in the case of statistical significance of the individual factors and their interaction. ^#^ Statistics: two-way ANOVA with six replicates (CT: cutting type; CL: cutting length; CT × CL: cutting type × cutting length interaction; the number indicates the F values, while the *p*-values are indicated as follows: * 0.05 > *p* > 0.01; *** 0.001 > *p*; ^NS^—not significant).

**Table 7:** Root length components [RLR (cm root length/g plant dry biomass): root length ratio; RMR (g root dry biomass/g plant dry biomass): root mass ratio; root fineness (cm root length/cm^3^ root volume); and root tissue density (g root dry biomass/cm^3^ root volume)] and root functional traits as root length distribution (average and standard deviation) among the different diameter classes [vFi (very fine): <0.05 mm; Fi (fine): 0.05 < D < 1 mm; and C (coarse): >1 mm)] (average ± standard deviation) of the root system of fig plants (cv. Dottato) of different cutting types (T1: tip portion of a one-year-old branch; T2: middle portion of a one-year-old branch; T3: basal portion of a one-year-old branch; T4: segment of a two-year-old branch) and lengths (A: 20 cm with 2–3 nodes; B: 10 cm with 3–4 nodes) 240 days after transplantation into a pot filled with a soil/peat/sand mixture.

Parameters	Statistics ^#^	Cutting Length (CL)	Cutting Type (CT)				CL Average
			T1	T2	T3	T4	
RLR (cm/g)	CT 1.58 ^NS^ CL 0.65 ^NS^ CT × CL 1.97 ^NS^	**A**	516 ± 146	633 ± 228	480 ± 103	506 ± 239	534
		**B**	350 ± 719	480 ± 117	743 ± 530	312 ± 199	470
		**CT average**	433	556	611	410	
RMR (g/g)	CT 1.93 ^NS^ CL 0.67 ^NS^ CT × CL 0.31 ^NS^	**A**	0.17 ± 0.06	0.19 ± 0.05	0.16 ± 0.03	0.14 ± 0.02	0.17
		**B**	0.15 ± 0.01	0.18 ± 0.05	0.16 ± 0.04	0.14 ± 0.03	0.16
		**CT average**	0.16	0.18	0.16	0.14	
Root fineness (cm/cm^3^)	CT 1.16 ^NS^ CL 4.49 * CT × CL 1.46 ^NS^	**A**	1114 ± 438	1730 ± 3279	1401 ± 281	1553 ± 400	1450 ^x^
		**B**	1135 ± 329	1098 ± 312	1332 ± 501	1196 ± 450	1190 ^y^
		**CT average**	1125	1410	1366	1375	
Root tissue density (g/cm^3^)	CT 0.69 ^NS^ CL 0.82 ^NS^ CT × CL 2.08 ^NS^	**A**	0.36 ± 0.11	0.53 ± 0.13	0.48 ± 0.05	0.47 ± 0.15	0.46
		**B**	0.48 ± 0.15	0.40 ± 0.06	0.33 ± 0.12	1.08 ± 1.35	0.42
		**CT average**	0.42	0.47	0.40	0.47	
vFi (cm)	CT 2.13 ^NS^ CL 5.76 * CT × CL 1.64 ^NS^	**A**	89,330 ± 34,036	139,197 ± 63,160	106,783 ± 34,063	120,060 ± 61,127	113,842 ^x^
		**B**	47,746 ± 12,258	79,605 ± 25,512	126,537 ± 73,671	59,424 ± 35,309	78,328 ^y^
		**CT average**	68,538 ^A^	109,400 ^A^	116,660 ^A^	89,742 ^A^	
Fi (cm)	CT 1.16 ^NS^ CL 4.49 * CT × CL 1.46 ^NS^	**A**	13,685 ± 4576	13,136 ± 5566	11,910 ± 3462	11,739 ± 2988	12,618 ^x^
		**B**	7079 ± 1475	12,384 ± 4586	14,862 ± 3142	9244 ± 9071	10,893 ^y^
		**CT average**	10,382	12,760	13,386	10,492	
C (cm)	CT 1.80 ^NS^ CL 0.07 ^NS^ CT × CL 0.07 ^NS^	**A**	701 ± 580	353 ± 244	1084 ± 550	666 ± 175	701
		**B**	446 ± 377	745 ± 272	939 ± 265	843 ± 1002	744
		**CT average**	574	549	1012	755	

Lowercase letters indicate significant differences among the means within columns (*p* < 0.05, Tukey’s test). Capital letters indicate significant differences among the means along the rows (*p* < 0.05, Tukey’s test). The letters are only reported in the case of statistical significance of the individual factors and their interaction. ^#^ Statistics: two-way ANOVA with six replicates (CT: cutting type; CL: cutting length; CT × CL: cutting type × cutting length interaction; the number indicates the F values, while the *p*-values are indicated as follows: * 0.05 > *p* > 0.01; ^NS^—not significant).

## Data Availability

Data are contained within the article.

## Supplementary Materials

The following supporting information can be downloaded at: https://www.mdpi.com/article/10.3390/plants14020160/s1. Table S1: Main physical and chemical characteristics soils soil used to prepare the soil:peat:sand; Table S2: Water content and distribution of the dry biomass (%) (average ± standard deviation) within the fig plants (cv. Dottato) of different cutting type (T1: tip portion of one-year-old branch; T2: middle portion of one-year-old branch; T3: basal portion of one-year-old branch; T4: segment of two-year-old branch) and length (A: 20 cm with 2–3 nodes; B: 10 cm with 3–4 nodes) after 60 days of transplanting in pot filled with perlite; Table S3: Water content and distribution of the dry biomass (%) (average ± standard deviation) within the fig plants (cv. Dottato) of different cutting type (T1: tip portion of one-year-old branch; T2: middle portion of one-year-old branch; T3: basal portion of one-year-old branch; T4: segment of two-year-old branch) and length (A: 20 cm with 2–3 nodes; B: 10 cm with 3–4 nodes) after 240 days of transplanting in pot filled with soil/peat/sand mixture; Table S4: Aboveground morphology (average and standard deviation) of the fig (cv. Dottato) plant and organs of different cutting type (T1: tip portion of one-year-old branch; T2: middle portion of one-year-old branch; T3: basal portion of one-year-old branch; T4: segment of two-year-old branch) and length (A: 20 cm with 2–3 nodes; B: 10 cm with 3–4 nodes) after 60 days of transplanting in pot filled with perlite; Table S5: Aboveground morphology (average and standard deviation) of the fig (cv. Dottato) plant and organs of different cutting type (T1: tip portion of one-year-old branch; T2: middle portion of one-year-old branch; T3: basal portion of one-year-old branch; T4: segment of two-year-old branch) and length (A: 20 cm with 2–3 nodes; B: 10 cm with 3–4 nodes) after 240 days of transplanting in pot filled with soil:peat:sand growing medium; Table S6: Principal Components of biometric and morphological parameters of the fig (cv. Dottato) plants growing for 60 days in pot filled with perlite (Varimax rotation and Kaiser normalization); Table S7: Principal Components of biometric and morphological parameters of the fig (cv. Dottato) plants growing for 240 days in pot filled with soil/peat/sand mixture (Varimax rotation and Kaiser normalization); Figure S1: Experimental scheme for the investigation of cutting type and length, and growth medium effects on rooting success, biomass yield and allocation, and root morphology in fig plants of cultivar “Dottato”.
