# Supplementary material for: Rooting, Growth, and Root Morphology of the Cuttings of Ficus carica L. (cv. “Dottato”): Cutting Types and Length and Growth Medium Effects
Source: Plants (Basel). 2025 Jan 8;14(2):160. doi: 10.3390/plants14020160 (PMC11771217; doi:10.3390/plants14020160)
Supplement: Supplementary file 1 [file plants-14-00160-s001.zip › plants-3369692-supplementary.pdf]

**Supplementary materials**

# Rooting, Growth and Root Morphology of the Cuttings of *Ficus carica* L. (cv. "Dottato"): Cutting Types and Length and Growth Medium Effects

**Rocco Mafrica[1*], Marcello Bruno[2], Vincenzo Fiozzo[1], Roberta Caridi[1], Agostino Sorgonà[1]**

1 [1] Department of AGRARIA, University "Mediterranea" of Reggio Calabria, Reggio Calabria, Italy

[2] Calabrian Agriculture Development Regional Agency (A.R.S.A.C.), Cosenza, Italy

* Correspondence: rocco.mafrica@unirc.it;Rocco Mafrica Tel. +39 0965 1694251)

**Table S1.** Main physical and chemical characteristics soils soil used to prepare the soil:peat:sand.

| Parameter | Value |
|---|---|
| Sand (%) | 80.5 |
| Silt (%) | 14.0 |
| Clay (%) | 5.5 |
| EC (dS $m^{-1}$) | 0.292 |
| pH [$H_2O$] (1:2.5) | 7.41 |
| Total carbonates (% $CaCO_3$) | 0 |
| Active carbonates (% $CaCO_3$) | 0 |
| Organic matter (%) | 2.74 |
| $N_{tot}$ (%) | 1.5 |
| C/N ratio | 10.9 |
| $P_{ava}$ (mg $kg^{-1}$) [Metodo Olsen] | 42 |
| $K_{ex}$ (mg $kg^{-1}$) | 109 |
| $Ca_{ex}$ (mg $kg^{-1}$) | 4353 |
| $Mg_{ex}$ (mg $kg^{-1}$) | 302 |
| $Na_{ex}$ (mg $kg^{-1}$) | 12 |
| Cation Exchange Capacity (meq 100 $g^{-1}$) | 17.3 |
| Exchangeable sodium percentage (ESP) (%) | 0.30 |
| Sodium Adsorpion Ratio (SAR) (%) | 0.02 |
| $Fe_{ava}$ (mg $kg^{-1}$) | 15 |
| $Mn_{ava}$ (mg $kg^{-1}$) | 10.44 |
| $Zn_{ava}$ (mg $kg^{-1}$) | 1.03 |

**Table S2**. Water content and distribution of the dry biomass (%) (average ± standard deviation) within the fig plants (cv. Dottato) of different cutting type (T1: tip portion of one-year-old branch; T2: middle portion of one-year-old branch; T3: basal portion of one-year-old branch; T4: segment of two-year-old branch) and length (A: 20 cm with 2-3 nodes; B: 10 cm with 3- 4 nodes) after 60 days of transplanting in pot filled with perlite.

| **Parameters** | **Statistics**[#] | **Cutting length (CL)** | **Cutting type (CT)** | | | | **CL average** |
|---|---|---|---|---|---|---|---|
| | | | **T1** | **T2** | **T3** | **T4** | |
| Plant water content (%) | CT 6.02** | **A** | 75.3$^{bc}$ ±3.57 | 74.47$^{bc}$ ±1.51 | 75.39$^{bc}$ ±2.23 | 75.48$^{bc}$ ±1.80 | 75.16 |
| | CL 2.59$^{NS}$ | **B** | 79.82$^{a}$ ±2.39 | 77.58$^{ab}$ ±1.49 | 75.56$^{bc}$ ±2.59 | 71.88$^{c}$ ±1.76 | 76.21 |
| | CTxCL 7.57*** | **CT average** | 77.56$^{A}$ | 76.03$^{AB}$ | 75.47$^{AB}$ | 73.68$^{B}$ | |
| Shoot axes water content (%) | CT 11.21*** | **A** | 73.72 ±3.62 | 70.51 ±1.38 | 70.22 ±2.53 | 68.51 ±6.89 | 70.74$^{Y}$ |
| | CL 17.87*** | **B** | 83.20 ±1.22 | 77.84 ±4.99 | 73.74 ±4.94 | 68.87 ±4.92 | 75.91$^{X}$ |
| | CTxCL 2.78NS | **CT average** | 78.46$^{A}$ | 74.17$^{AB}$ | 71.98$^{BC}$ | 68.69$^{C}$ | |
| Leaf water content (%) | CT 7.36*** | **A** | 83.92 ±2.50 | 82.95 ±3.03 | 84.82 ±3.95 | 81.68 ±3.00 | 83.34 |
| | CL 2.00$^{NS}$ | **B** | 83.60 ±2.77 | 82.71 ±1.73 | 84.69 ±2.39 | 77.89 ±1.89 | 82.22 |
| | CTxCL 1.27$^{NS}$ | **CT average** | 83.76$^{A}$ | 82.83$^{A}$ | 84.76$^{A}$ | 79.79$^{B}$ | |
| Cutting water content (%) | CT 8.57*** | **A** | 67.09 ±1.83 | 64.34 ±1.45 | 64.67 ±0.79 | 72.85 ±3.38 | 67.24 |
| | CL 2.79$^{NS}$ | **B** | 73.25 ±2.89 | 69.27 ±3.49 | 65.36 ±2.97 | 66.69 ±4.58 | 68.64 |
| | CTxCL 10.93*** | **CT average** | 70.17$^{A}$ | 66.80$^{BC}$ | 65.07$^{C}$ | 69.77$^{AB}$ | |
| Root water content (%) | CT 0.97$^{NS}$ | **A** | 81.28 ±14.92 | 83.95 ±8.92 | 83.97 ±2.99 | 80.95 ±5.7) | 82.54 |
| | CL 0.74$^{NS}$ | **B** | 90.07 ±1.45 | 84.35 ±8.57 | 83.41 ±6.21 | 79.97 ±3.68 | 84.45 |
| | CTxCL 1.08$^{NS}$ | **CT average** | 85.67 | 84.15 | 83.69 | 80.47 | |
| Shoot dry biomass (%) | CT 4.39** | **A** | 8.74 ±1.21 | 8.17 ±1.48 | 10.24 ±2.61 | 10.99 ±3.79 | 9.54$^{X}$ |
| | CL 4.58* | **B** | 5.39 ±1.34 | 7.65 ±3.57 | 7.61 ±3.00 | 10.78 ±3.25 | 7.86$^{Y}$ |
| | CTxCL 0.97$^{NS}$ | **CT average** | 7.07$^{B}$ | 7.91$^{AB}$ | 8.93$^{AB}$ | 10.89$^{A}$ | |
| Leaf dry biomass (%) | CT 1.57$^{NS}$ | **A** | 20.74 ±8.67 | 24.58 ±6.53 | 24.69 ±6.13 | 21.21 ±5.11 | 22.81$^{Y}$ |
| | CL 14.78*** | **B** | 33.23 ±6.73 | 32.91 ±3.25 | 23.84 ±4.19 | 27.65 ±5.27 | 29.41$^{X}$ |
| | CTxCL 2.63$^{NS}$ | **CT average** | 26.99 | 28.75 | 24.27 | 24.43 | |
| Cutting dry biomass (%) | CT 1.22$^{NS}$ | **A** | 61.94 ±6.66 | 57.61 ±6.48 | 58.77 ±4.87 | 60.38 ±9.32 | 59.68 |
| | CL 2.97$^{NS}$ | **B** | 56.67 ±6.56 | 52.54 ±6.15 | 60.94 ±6.30 | 55.19 ±6.46 | 56.34 |
| | CTxCL 0.90$^{NS}$ | **CT average** | 59.31 | 55.08 | 59.86 | 57.79 | |
| Root dry biomass (%) | CT 0.37$^{NS}$ | **A** | 8.56 ±3.27 | 9.62 ±7.51 | 6.27 ±1.77 | 7.40 ±5.00 | 7.97 |
| | CL 1.75$^{NS}$ | **B** | 4.69 ±1.46 | 6.87 ±3.51 | 7.59 ±3.18 | 6.36 ±4.24 | 6.38 |
| | CTxCL 0.88$^{NS}$ | **CT average** | 6.63 | 8.25 | 6.94 | 6.87 | |

Lowercase letters indicated significant difference among the means within columns ($p$<0.05, Tukey's test). Capital letters indicated significant difference among the means along the rows ($p$<0.05, Tukey's test). The letters are only reported in the case of statistical significance of the individual factors and their interaction. [#]Statistics: two-way ANOVA with $N = 6$ (CT: cutting type; CL: cutting length; CT x CL: cutting type x cutting length interaction; * 0,05>$P$<0,01; ** 0,01>$P$<0,001; *** 0,001>$P$; NS not significant.

**Table S3**. Water content and distribution of the dry biomass (%) (average ± standard deviation) within the fig plants (cv Dottato) of different cutting type (T1: tip portion of one-year-old branch; T2: middle portion of one-year-old branch; T3: basal portion of one-year-old branch; T4: segment of two-year-old branch) and length (A: 20 cm with 2-3 nodes; B: 10 cm with 3- 4 nodes) after 240 days of transplanting in pot filled with soil/peat/sand mixture.

| **Parameters** | **Statistics**[#] | **Cutting length** | **Cutting type (CT)** | **CL average** |
|---|---|---|---|---|

| | | (CL) | T1 | T2 | T3 | T4 | |
|---|---|---|---|---|---|---|---|
| Plant water content (%) | CT 19.37*** | **A** | 79.91 ±0.77 | 78.47 ±0.45 | 78.97 ±0.10 | 77.82 ±0.78 | 78.79$^{Y}$ |
| | CL 8.55** | **B** | 80.23 ±0.25 | 79.39 ±0.62 | 79.01 ±0.52 | 78.57 ±0.56 | 79.31$^{X}$ |
| | CTxCL 1.30$^{NS}$ | **CT average** | 80.07$^{A}$ | 78.93$^{B}$ | 78.99$^{B}$ | 78.19$^{C}$ | |
| Shoot axes water content (%) | CT 35.08*** | **A** | 74.61 ±0.94 | 72.73 ±1.06 | 72.28 ±0.91 | 70.18 ±0.12 | 72.45$^{Y}$ |
| | CL 34.05*** | **B** | 75.94 ±0.50 | 74.25 ±0.59 | 72.66 ±0.72 | 73.11 ±1.25 | 73.99$^{X}$ |
| | CTxCL 3.97* | **CT average** | 75.28$^{A}$ | 73.49$^{B}$ | 72.47$^{C}$ | 71.65$^{C}$ | |
| Leaf water content (%) | CT 1.27$^{NS}$ | **A** | 80.08 ±1.40 | 82.12 ±3.85 | 85.21 ±2.78 | 80.32 ±2.34 | 81.93 |
| | CL 0.98$^{NS}$ | **B** | 83.20 ±1.35 | 79.43 ±1.98 | 81.67 ±1.26 | 82.45 ±1.43 | 81.69 |
| | CTxCL 0.78$^{NS}$ | **CT average** | 81.64 | 80.77 | 83.44 | 81.38 | |
| Cutting water content (%) | CT 5.98** | **A** | 69.33 ±1.50 | 67.91 ±0.60 | 68.77 ±0.74 | 67.25 ±2.31 | 68.32$^{Y}$ |
| | CL 5.03* | **B** | 70.44 ±0.90 | 70.19 ±1.44 | 69.19 ±1.76 | 67.38 ±0.99 | 69.31$^{X}$ |
| | CTxCL 1.18$^{NS}$ | **CT average** | 69.89$^{A}$ | 69.06$^{A}$ | 68.98$^{AB}$ | 67.32$^{B}$ | |
| Root water content (%) | CT 2.67$^{NS}$ | **A** | 83.32 ±2.66 | 80.11 ±1.01 | 82.22 ±0.46 | 81.09 ±2.50 | 81.69 |
| | CL 0.00$^{NS}$ | **B** | 82.26 ±1.82 | 81.35 ±2.16 | 82.06 ±1.64 | 81.06 ±1.33 | 81.69 |
| | CTxCL 0.66$^{NS}$ | **CT average** | 82.79 | 80.73 | 82.14 | 81.08 | |
| Shoot dry biomass (%) | CT 12.38*** | **A** | 28.10 ±2.67 | 28.35 ±2.26 | 31.60 ±1.62 | 34.29 ±1.62 | 30.59 |
| | CL 1.99$^{NS}$ | **B** | 28.52 ±0.75 | 28.86 ±1.94 | 30.79 ±1.78 | 30.87 ±1.55 | 29.76 |
| | CTxCL 2.48$^{NS}$ | **CT average** | 28.32$^{B}$ | 28.61$^{B}$ | 31.2$^{A}$ | 32.58$^{A}$ | |
| Leaf dry biomass (%) | CT 6.37** | **A** | 43.53 ±5.39 | 38.80 ±3.79 | 40.47 ±2.07 | 38.67 ±1.76 | 40.37$^{Y}$ |
| | CL 5.89* | **B** | 47.61 ±1.87 | 41.80 ±3.45 | 41.82 ±3.71 | 40.57 ±3.16 | 42.95$^{X}$ |
| | CTxCL 0.32$^{NS}$ | **CT average** | 45.75$^{A}$ | 41.15$^{B}$ | 40.30$^{B}$ | 39.62$^{B}$ | |
| Cutting dry biomass (%) | CT 4.88** | **A** | 11.01 ±2.71 | 14.15 ±1.54 | 11.50 ±1.23 | 12.94 ±2.89 | 12.40 |
| | CL 1.37$^{NS}$ | **B** | 9.34 ±1.67 | 11.31 ±1.37 | 11.82 ±1.84 | 14.07 ±2.50 | 11.64 |
| | CTxCL 1.94$^{NS}$ | **CT average** | 10.18$^{B}$ | 12.73$^{A}$ | 11.66$^{AB}$ | 13.51$^{A}$ | |
| Root dry biomass (%) | CT 1.92$^{NS}$ | **A** | 17.34 ±5.50 | 18.68 ±4.94 | 16.40 ±2.55 | 14.08 ±1.60 | 16.63 |
| | CL 0.67$^{NS}$ | **B** | 14.51 ±1.18 | 18.01 ±5.08 | 15.55 ±4.08 | 14.48 ±2.85 | 15.64 |
| | CTxCL 0.31$^{NS}$ | **CT average** | 15.92 | 18.34 | 15.98 | 14.28 | |

Capital letters indicated significant difference among the means along the rows ($p$<0.05, Tukey's test). The letters are only reported in the case of statistical significance of the individual factors and their interaction. [#]Statistics: two-way ANOVA with $N = 6$ (CT: cutting type; CL: cutting length; CT x CL: cutting type x cutting length interaction; * 0,05>$P$<0,01; ** 0,01>$P$<0,001; *** 0,001>$P$; NS not significant.

**Table S4.** Aboveground morphology (average and standard deviation) of the fig (cv Dottato) plant and organs of different cutting type (T1: tip portion of one-year-old branch; T2: middle portion of one-year-old branch; T3: basal portion of one-year-old branch; T4: segment of two-year-old branch) and length (A: 20 cm with 2-3 nodes; B: 10 cm with 3- 4 nodes) after 60 days of transplanting in pot filled with perlite.

| Parameters | Statistics[#] | Cutting length | Cutting type (CT) | | | | CL average |
|---|---|---|---|---|---|---|---|

| | | (CL) | T1 | T2 | T3 | T4 | |
|---|---|---|---|---|---|---|---|
| Shoot length (cm) | CT 33.89*** | **A** | 11.00 ±2.42 | 17.83 ±2.79 | 22.67 ±2.33 | 35.18 ±2.28 | 21.7$^{X}$ |
| | CL 7.78** | **B** | 8.00 ±0.45 | 15.50 ±2.59 | 18.58 ±1.67 | 27.23 ±2.20 | 17.3$^{Y}$ |
| | CTxCL 0.65NS | **CT average** | 9.5$^{C}$ | 16.7$^{BC}$ | 20.6$^{B}$ | 31.2$^{A}$ | |
| Total leaf area ($cm^2$) | CT 5.29** | **A** | 626 ±139 | 912 ±121 | 1053 ±175 | 889 ±96 | 870$^{X}$ |
| | CL 8.58** | **B** | 403 ±56 | 643 ±73 | 829 ±98 | 700 ±57 | 644$^{Y}$ |
| | CTxCL 0.05NS | **CT average** | 515$^{C}$ | 778$^{B}$ | 941$^{A}$ | 794$^{B}$ | |
| Leaf numbers (n) | CT 7.57** | **A** | 13.5 ±1.3 | 14.8 ±2.3 | 15.2 ±1.0 | 22.8 ±2.2 | 16.6$^{X}$ |
| | CL 1.45NS | **B** | 11.5 ±1.1 | 16.8 ±2.6 | 14.7 ±1.1 | 17.7 ±0.7 | 15.2$^{X}$ |
| | CTxCL 1.62NS | **CT average** | 12.5$^{C}$ | 15.8$^{B}$ | 14.9$^{B}$ | 20.3$^{A}$ | |
| Average leaf area ($cm^2$) | CT 4.07* | **A** | 44.7 ±7.4 | 64.4 ±8.4 | 74.3 ±17.2 | 41.6 ±7.1 | 56.2$^{X}$ |
| | CL 4.45* | **B** | 35.1 ±4.1 | 42.0 ±6.7 | 57.1 ±6.2 | 39.8 ±3.2 | 43.5$^{Y}$ |
| | CTxCL 0.55NS | **CT average** | 39.9$^{B}$ | 53.2$^{AB}$ | 65.7$^{A}$ | 40.7$^{B}$ | |

Capital letters indicated significant difference among the means along the rows ($p$<0.05, Tukey's test). The letters are reported only in the case of significance of the individual factors and their interaction. #Statistics: two-way ANOVA with six replicates (CT: cutting type; CL: cutting length; CT x CL: cutting type x cutting length interaction; the number indicate the F values, while the $P$ values are indicated as follow: * 0,05>$P$<0,01; ** 0,01>$P$<0,001; *** 0,001>$P$; NS not significant.

**Table S5.** Aboveground morphology (average and standard deviation) of the fig (cv Dottato) plant and organs of different cutting type (T1: tip portion of one-year-old branch; T2: middle portion of one-year-old branch; T3: basal portion of one-year-old branch; T4: segment of two-year-old branch) and length (A: 20 cm with 2-3 nodes; B: 10 cm with 3- 4 nodes) after 240 days of transplanting in pot filled with soil:peat:sand growing medium.

| **Parameters** | **Statistics#** | **Cutting length (CL)** | **Cutting type (CT)** | | | | **CL average** |
|---|---|---|---|---|---|---|---|
| | | | **T1** | **T2** | **T3** | **T4** | |
| Shoot length (cm) | CT 46.24*** | **A** | 154 ±2 | 173 ±4 | 182 ±4 | 190 ±2 | 175$^{X}$ |
| | CL 64.13*** | **B** | 137 ±3 | 154 ±4 | 165 ±3 | 172 ±3 | 157$^{Y}$ |
| | CTxCL 0.08NS | **CT average** | 146$^{C}$ | 163$^{B}$ | 174$^{A}$ | 181$^{A}$ | |
| Total leaf area ($cm^2$) | CT 19.05*** | **A** | 13399 ±218 | 14172 ±204 | 15525 ±449 | 15718 ±328 | 14704$^{X}$ |
| | CL 45.47*** | **B** | 11724 ±332 | 12433 ±332 | 13206 ±444 | 14495 ±499 | 12965$^{Y}$ |
| | CTxCL 0.76NS | **CT average** | 12562$^{B}$ | 13303$^{B}$ | 14366$^{A}$ | 15106$^{A}$ | |
| Leaf numbers (n) | CT 21.02*** | **A** | 42 ±0.7 | 44 ±0.6 | 48 ±1.4 | 49 ±1.0 | 46$^{X}$ |
| | CL 35.40*** | **B** | 36 ±1.0 | 39 ±1.0 | 41 ±1.4 | 45 ±1.5 | 40$^{Y}$ |
| | CTxCL 0.65NS | **CT average** | 39$^{B}$ | 41$^{B}$ | 45$^{A}$ | 47$^{A}$ | |
| Average leaf area ($cm^2$) | CT 0.97NS | **A** | 319 ±25 | 322 ±20 | 323 ±22 | 320 ±23 | 319 |
| | CL 0.85NS | **B** | 325 ±18 | 318 ±26 | 322 ±27 | 322 ±29 | 324 |
| | CTxCL 0.55NS | **CT average** | 322 | 324 | 319 | 321 | |

Capital letters indicated significant difference among the means along the rows ($p$<0.05, Tukey's test). The letters are reported only in the case of significance of the individual factors and their interaction. #Statistics: two-way ANOVA with six replicates (CT: cutting type; CL: cutting length; CT x CL: cutting type x cutting length interaction; the number indicate the F values, while the $P$ values are indicated as follow: *** 0,001>$P$; NS not significant.

**Table S6.** Principal Components of biometric and morphological parameters of the fig (cv Dottato) plants growing for 60 days in pot filled with perlite (Varimax rotation and Kaiser normalization).

| **Parameters** | **PC1** | **PC2** | **PC3** |
|---|---|---|---|
| Leaf fresh biomass | **.879** | .149 | .239 |
| Root fresh biomass | .289 | **.831** | .321 |
| Root dry biomass | .166 | **.909** | .282 |
| Leaf surface area | **.974** | -.001 | .107 |
| Average leaf surface area | **.873** | .125 | -.172 |
| Root diameter | .038 | .204 | **.899** |
| RMR | -.082 | **.947** | .107 |

| | | | |
|---|---|---|---|
| Root fineness | .063 | .278 | **.858** |

**Table S7.** Principal Components of biometric and morphological parameters of the fig (cv Dottato) plants growing for 240 days in pot filled with soil/peat/sand mixture (Varimax rotation and Kaiser normalization).

| Parameters | PC1 | PC2 | PC3 |
|---|---|---|---|
| Shoot fresh biomass | **0.986** | 0.027 | 0.061 |
| Shoot dry biomass | **0.954** | 0.096 | 0.107 |
| Leaf fresh biomass | **0.985** | 0.062 | 0.091 |
| Leaf dry biomass | **0.985** | 0.062 | 0.091 |
| Root dry biomass | 0.284 | 0.184 | **0.935** |
| Shoot length | **0.904** | 0.223 | 0.155 |
| Leaf number | **0.985** | 0.062 | 0.091 |
| Leaf surface area | **0.985** | 0.062 | 0.091 |
| Root length | 0.197 | **0.949** | 0.236 |
| RLR | -0.085 | **0.988** | -0.008 |
| RMR | -0.184 | 0.086 | **0.972** |
| vFi | 0.205 | **0.951** | 0.213 |
| Root fresh biomass | 0.318 | 0.159 | **0.916** |

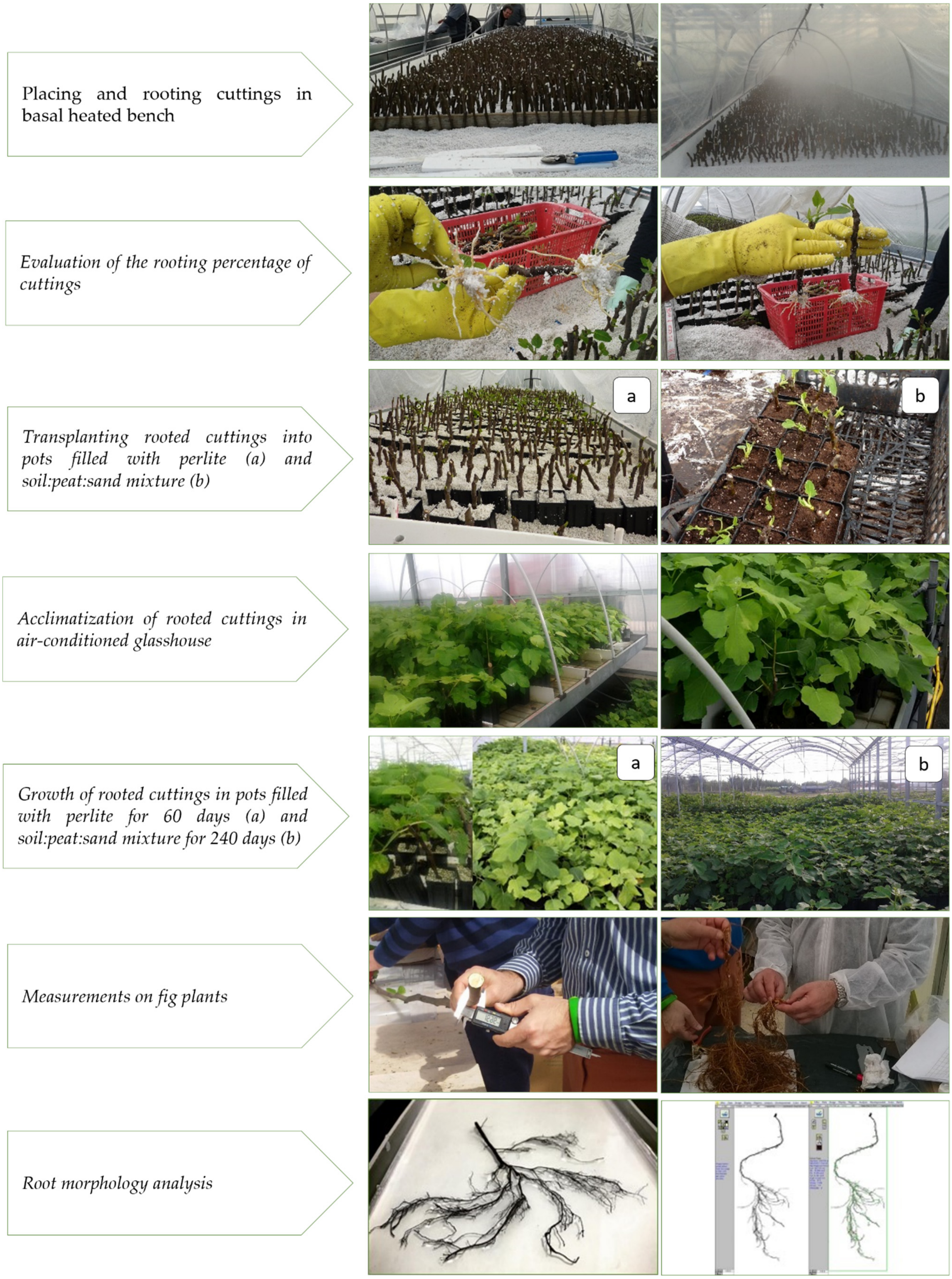


**Figure S1.** Experimental scheme for the investigation of cutting type and length, and growth medium effects on rooting success, biomass yield and allocation, and root morphology in fig plants of cultivar “Dottato”.
